# Supplementary material for: Asynchronous magnetic resonance elastography: Shear wave speed reconstruction using noise correlation of incoherent waves
Source: Magn Reson Med. 2022 Oct 27;89(3):990–1001. doi: 10.1002/mrm.29502 (PMC9792433; doi:10.1002/mrm.29502)
Supplement: Supplementary file 1 — DATA S1 MATLAB code used to generate the numerical simulations of Figure 1 [file MRM-89-990-s002.zip › k-Wave/helpfiles/makeArc.html]

makeArc :: Functions (k-Wave)


# makeArc

Create a binary map of an arc within a 2D grid.

## Syntax

```
arc = makeArc(grid_size, arc_pos, radius, diameter, focus_pos)
arc = makeArc(grid_size, arc_pos, radius, diameter, focus_pos, ...)
```

## Description

`makeArc` creates a binary map of an arc within a two-dimensional grid. The arc position is denoted by 1's in the matrix with 0's elsewhere. The arc is found using the mid-point circle algorithm, and is simply connected so that any grid point on the arc will have at most 2 neighbours.

The midpoint of the arc is set by `arc_pos`. The orientation of the arc is set by `focus_pos`, which corresponds to any point on the axis of the arc (note, this must not be equal to `arc_pos`). It is assumed that the arc angle is equal to or less than pi radians. If the radius is set to `inf`, a line is generated.

## Examples

```
% define parameters
grid_size = [128, 128];
arc_pos   = [32, 32];
radius    = 60;
diameter  = 61;
focus_pos = grid_size;

% create arc
arc = makeArc(grid_size, arc_pos, radius, diameter, focus_pos, 'Plot', true);
```

## Inputs

|  |  |
| --- | --- |
| `grid_size` | size of the 2D grid given as a three element vector [Nx, Ny] [grid points] |
| `bowl_pos` | midpoint of the arc given as a two element vector [ax, ay] [grid points] |
| `radius` | radius of curvature of the arc [grid points] |
| `diameter` | aperture diameter (length of line connecting arc endpoints) [grid points] |
| `focus_pos` | any point on the beam axis of the arc given as a two element vector [fx, fy] [grid points] |

## Optional Inputs

Optional 'string', value pairs that may be used to modify the default computational settings.

| Input | Valid Settings | Default | Description |
| --- | --- | --- | --- |
| `'Plot'` | *(Boolean scalar)* | `false` | Boolean controlling whether the arc is plotted using `imagesc`. |

## Outputs

|  |  |
| --- | --- |
| `arcs` | 2D binary map of arcs |
| `arc` | 2D labelled matrix of arcs |

## See Also

`makeBowl`, `makeLine`, `makeMultiArc`
